# Dihomo-*γ*-Linolenic Acid (20:3n-6)—Metabolism, Derivatives, and Potential Significance in Chronic Inflammation

**DOI:** 10.3390/ijms24032116

**Published:** 2023-01-20

**Authors:** Anne-Mari Mustonen, Petteri Nieminen

**Affiliations:** 1 Department of Environmental and Biological Sciences, Faculty of Science, Forestry and Technology, University of Eastern Finland, P.O. Box 111, FI-80101 Joensuu, Finland; 2 Faculty of Health Sciences, Institute of Biomedicine, School of Medicine, University of Eastern Finland, P.O. Box 1627, FI-70211 Kuopio, Finland

**Keywords:** DGLA, dihomo-*γ*-linolenic acid, inflammation, lipid mediator, n-6 PUFA, oxylipin

## Abstract

Dihomo-*γ*-linolenic acid (DGLA) has emerged as a significant molecule differentiating healthy and inflamed tissues. Its position at a pivotal point of metabolic pathways leading to anti-inflammatory derivatives or via arachidonic acid (ARA) to pro-inflammatory lipid mediators makes this n-6 polyunsaturated fatty acid (PUFA) an intriguing research subject. The balance of ARA to DGLA is probably a critical factor affecting inflammatory processes in the body. The aim of this narrative review was to examine the potential roles of DGLA and related n-6 PUFAs in inflammatory conditions, such as obesity-associated disorders, rheumatoid arthritis, atopic dermatitis, asthma, cancers, and diseases of the gastrointestinal tract. DGLA can be produced by cultured fungi or be obtained via endogenous conversion from *γ*-linolenic acid (GLA)-rich vegetable oils. Several disease states are characterized by abnormally low DGLA levels in the body, while others can feature elevated levels. A defect in the activity of ∆6-desaturase and/or ∆5-desaturase may be one factor in the initiation and progression of these conditions. The potential of GLA and DGLA administrations as curative or ameliorating therapies in inflammatory conditions and malignancies appears modest at best. Manipulations with ∆6- and ∆5-desaturase inhibitors or combinations of long-chain PUFA supplements with n-3 PUFAs could provide a way to modify the body’s DGLA and ARA production and the concentrations of their pro- and anti-inflammatory mediators. However, clinical data remain scarce and further well-designed studies should be actively promoted.

## 1. Introduction

**Figure 1:**
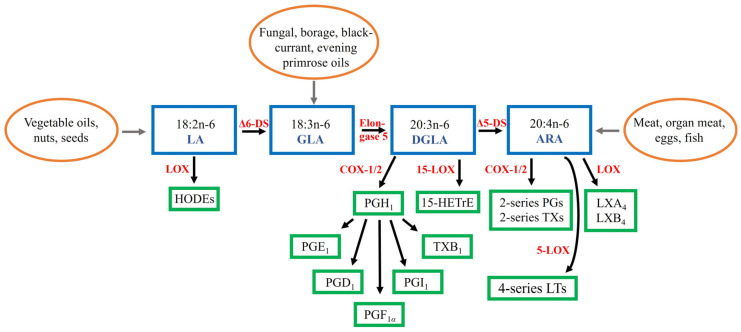
Pathways of conversion of n-6 polyunsaturated fatty acids and their lipid mediator derivatives [1,4,5]. Note that not all enzymes are named, or derivatives shown. ARA = arachidonic acid, COX = cyclooxygenase, DGLA = dihomo-*γ*-linolenic acid, DS = desaturase, GLA = *γ*-linolenic acid, 15-HETrE = 15-(S)-hydroxy-8,11,13-eicosatrienoic acid, HODE = hydroxyoctadecadienoic acid, LA = linoleic acid, LOX = lipoxygenase, LT = leukotriene, LX = lipoxin, PG = prostaglandin, TX = thromboxane.

Dihomo-*γ*-linolenic acid (DGLA, 20:3n-6) is a polyunsaturated fatty acid (PUFA) that is usually present in low proportions in mammals but has recently emerged as a significant molecule differentiating healthy and inflamed tissues. The general overview of the biosynthesis of DGLA, other n-6 PUFAs, and related lipid mediator derivatives is represented in Figure 1. The precursor of DGLA, *γ*-linolenic acid (GLA, 18:3n-6), can be obtained in small amounts from human milk and organ meats [1]. However, seed oils, such as borage *Borago officinalis* oil (approximately 18–26 wt-% of GLA), blackcurrant *Ribes nigrum* oil (15–20%), and evening primrose *Oenothera biennis* oil (7–10%), as well as fungal oils (23–26%) are rich sources of GLA. It is also formed by ∆6-desaturase, which is encoded by fatty acid desaturase 2 (*FADS2*), from linoleic acid (LA, 18:2n-6), the dietarily essential n-6 PUFA [2,3]. LA is considered the principal PUFA in most Western diets being present in vegetable oils, nuts, and seeds [4].

GLA is rapidly elongated (*ELOVL5*) to DGLA, which can be further desaturated by ∆5-desaturase (*FADS1*) to arachidonic acid (ARA, 20:4n-6) [1,2]. However, only a small fraction of DGLA is converted to ARA due to the limited activity of ∆5-desaturase and, thus, in many cell types DGLA instead of ARA becomes accumulated after dietary GLA supplementation. DGLA is metabolized via the cyclooxygenase (COX-1 and COX-2) pathway to 1-series prostaglandins (PGs), particularly PGE_1_, or converted via the 15-lipoxygenase (LOX) pathway into 15-(S)-hydroxy-8,11,13-eicosatrienoic acid (15-HETrE). These two metabolites have been shown to suppress inflammation, promote vasodilation, lower blood pressure, inhibit smooth muscle cell proliferation, and to exert anti-neoplastic activities. The inhibition of vascular smooth muscle cell (VSMC) proliferation would be especially significant, as agents that reduce their migration and proliferation also retard the formation of the typical atherosclerotic plaque. Furthermore, both PGE_1_ and 15-HETrE antagonize the synthesis of ARA-derived lipid mediators.

Dietary ARA can be obtained from meats, organ meats, and eggs [4]. It is converted into 2-series PGs and thromboxanes and 4-series leukotrienes (LTs) that tend to be pro-inflammatory in several cell types and disease states [2]. ARA is usually the major PUFA in cellular membranes of inflammatory cells [4]. Increased dietary ARA intake leads to elevated ARA levels in membrane phospholipids (PLs) and increased production of ARA-derived lipid mediators. PGE_2_ is the pivotal oxylipin from ARA that has both pro- and anti-inflammatory actions [3]. Regarding synovial joints, it is involved in the pathogeneses of inflammatory joint diseases [6] while, in the respiratory system, PGE_2_ increases the relaxation of airway smooth muscle and inhibits the release of mast cell mediators and the recruitment of inflammatory cells [7]. ARA can also be converted to lipoxin A_4_ (LXA_4_) that belongs to specialized pro-resolving mediators (SPMs) and exhibits actions similar to PGE_1_, as both are anti-inflammatory, vasodilatory, platelet anti-aggregatory, cytoprotective, genoprotective, and anti-diabetic molecules [3]. It has been proposed that ARA would be the principal PUFA responsible for the switchover from the pro-inflammatory to the anti-inflammatory state by the formation of LXA_4_ instead of PGE_2_. SPMs (resolvins, protectins, and maresins) derived from n-3 PUFAs eicosapentaenoic acid (EPA, 20:5n-3) and docosahexaenoic acid (DHA, 22:6n-3) could serve more as supporters of the resolution process initiated by LXA_4_.

Similar to ARA, DGLA is located in cell membrane PLs and released as free FAs by phospholipase A_2_ [2]. The beneficial effects of DGLA presumably result from both the anti-inflammatory properties of its derivatives and the ability to compete with ARA in the synthesis of pro-inflammatory ARA mediators. The balance of ARA to DGLA is probably a critical factor affecting inflammatory processes in the body, and an increase in DGLA relative to ARA levels can attenuate the biosynthesis of 2-series PGs and 4-series LTs [1]. In vitro and in vivo animal experiments have demonstrated potentially beneficial effects of dietary GLA, the DGLA precursor, on inflammatory conditions, such as rheumatoid arthritis (RA) and atopic eczema, but clinical data are less convincing [8,9]. It is now known that the earlier view of n-6 PUFAs and their derivatives being generally pro-inflammatory and, thus, harmful was an oversimplification. In fact, ARA and LA have also been linked to reduced inflammation [4]. This narrative review was undertaken to examine the potential roles of DGLA and related n-6 PUFAs in low-grade and overt inflammation. While the theoretical background points to health benefits, many unresolved issues remain regarding how these FAs are used in the synthesis of lipid mediators, the complex balance of their pro- and anti-inflammatory roles, and, especially, the potential of therapeutic applications.

## 2. Literature Search

The PubMed (https://pubmed.ncbi.nlm.nih.gov/) and Web of Science databases (https://www.webofscience.com/wos/woscc/basic-search) were searched for relevant literature with the keywords “dihomo-*γ*-linolenic acid”, “DGLA”, “20:3n-6”, and “inflammation”. The search was conducted in the autumn of 2022 until 21 December and was limited to English-language original research articles and reviews. One author (A.-M.M.) screened the titles and abstracts of potential literature and determined their eligibility, and the bibliographies of relevant articles were examined for additional references. A total of 77 papers were included in this narrative review. The intention was not to cite all relevant literature but to select studies that would help us (i) to gain an up-to-date view of the recent research on the potential associations of DGLA to inflammation and (ii) to critically assess if it would be possible to develop novel diagnostic, prognostic, and therapeutic tools based on these phenomena.

## 3. DGLA in Inflammatory Conditions

Plasma DGLA levels have been directly associated with several inflammatory markers, such as high-sensitivity C-reactive protein and cytokines [10]. As low-grade inflammation is a well-recognized risk factor for the development of chronic diseases, including type 2 diabetes (T2D) and atherosclerosis, n-6 PUFA profiles could serve as an early indicator for the development of future complications. This chapter discusses the associations between DGLA levels and inflammatory diseases. Our aim is to introduce selected examples of disease states that are characterized by disturbed n-6 PUFA metabolism, especially those featured with altered DGLA levels (Table 1).

**Figure 2:**
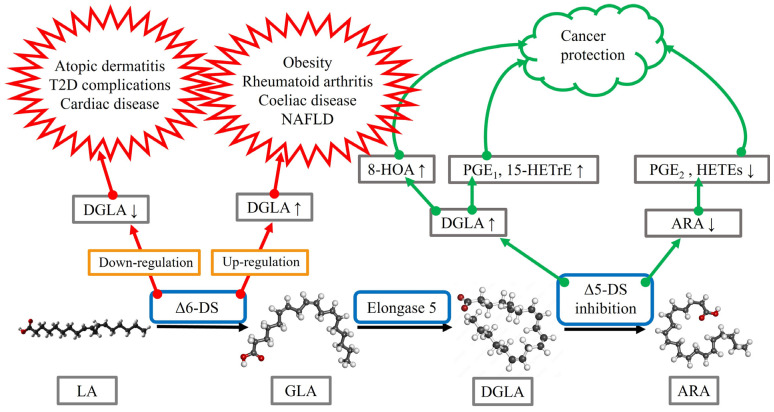
A summary of the potential benefits and hazards of desaturase manipulation based on the current literature review. ARA = arachidonic acid, DGLA = dihomo-*γ*-linolenic acid, DS = desaturase, GLA = *γ*-linolenic acid, HETE = hydroxyeicosatetraenoic acid, 15-HETrE = 15-(S)-hydroxy-8,11,13-eicosatrienoic acid, 8-HOA = 8-hydroxyoctanoic acid, LA = linoleic acid, NAFLD = non-alcoholic fatty liver disease, PG = prostaglandin. Open-source images provided by Creative Commons and Adobe Stock (https://commons.wikimedia.org/wiki/File:Linoleic-acid-from-xtal-1979-3D-balls.png; https://commons.wikimedia.org/wiki/File:Gamma_linolenic_acid.png; https://as2.ftcdn.net/v2/jpg/01/21/79/17/1000_F_121791777_FxcSPoTFG0SAP9oEmm4tR57IgsB9fQfl.jpg; https://upload.wikimedia.org/wikipedia/commons/5/50/Arachidonic_acid2.png; all image sites accessed 21 November 2022).

Obesity is associated with low-grade inflammation, and obese subjects are at increased risk of, for instance, T2D, liver lipidosis, cardiovascular diseases, osteoarthritis (OA), respiratory complications, and cancer [11]. The levels of DGLA, but not ARA, were significantly higher in overweight or obese subjects compared to controls when measured from plasma total lipids, PLs, and cholesteryl esters. As ARA/DGLA ratios (surrogate for Δ5-desaturase activity) were reduced and DGLA/LA ratios (surrogate for Δ6-desaturase activity) elevated, the underlying cause for the disturbed n-6 PUFA metabolism in obesity could be related to increased Δ6-desaturase activity in parallel with decreased Δ5-desaturase activity. Abnormal desaturase levels can accompany several obesity-associated disorders, as discussed below (Figure 2). The dietary intervention of obese subjects with a very low-carbohydrate diet for 8 weeks decreased their serum DGLA concentrations together with significant improvements of clinical characteristics and metabolic markers [12].

Serum DGLA levels were also elevated in overweight/obese subjects with T2D compared to metabolically healthy overweight/obese subjects [12]. In patients with T2D, serum DGLA levels showed positive correlations with waist circumference, body fat-%, body mass index, and homeostatic model assessment of insulin resistance (HOMA-IR) [13]. During pregnancy, plasma DGLA was also positively associated with the levels of HOMA-IR and C-peptide, well-known risk factors for the development of gestational diabetes mellitus [14]. Higher concentrations of fasting whole blood DGLA, ARA, and several n-3 PUFAs were associated with an increased risk of T2D [15], and the baseline serum concentrations of DGLA could predict the development of future metabolic syndrome in overweight/obese subjects [12]. Complications of T2D have also exhibited altered n-6 PUFA metabolism. Patients with diabetic nephropathy had reduced DGLA levels in plasma PLs [16], and serum DGLA was inversely associated with the presence of diabetic retinopathy, on which it may have ameliorative influence via its oxidative metabolites PGE_1_ and 15-HETrE [17]. A GLA–lipoic acid (an antioxidant) conjugate improved neuropathy in a diabetic rat model [18]. In T2D patients with vasculogenic erectile dysfunction, indices of Δ6-desaturase and elongase activities were lower than in the control group together with significant decreases in the erythrocyte membrane DGLA levels [19]. Erection could be partly mediated by the DGLA-derived PGE_1_ that promotes the relaxation of penile VSMCs via cAMP. Elevated DGLA in plasma PLs has been identified as a potential biomarker for the insulin resistance complication of polycystic ovary syndrome [20].

**Table 1:** Selected data regarding dihomo-*γ*-linolenic acid (DGLA) or, in some cases, *γ*-linolenic acid (GLA) and their potential associations to inflammatory disease risk or treatment.

Condition/Disease	Finding	Reference
Obesity	Increased DGLA levels in overweight/obese subjects	[11]
	High maternal DGLA levels predict childhood body fat	[21]
Type 2 diabetes	Direct relationship between DGLA and HOMA-IR	[13]
(T2D)	Increased DGLA levels in overweight/obese subjects with T2D	[12]
	High DGLA levels associated with an increased risk of T2D	[15]
	Inverse relationship with complications of T2D	[16,17,19]
Cardiovascular	DGLA supplement reduces atherosclerosis development	[22]
diseases	Low DGLA levels worsen disease prognosis	[23,24]
Hepatic diseases	High DGLA levels associated with disease development	[25,26,27]
	High maternal DGLA levels predict childhood lipidosis	[28]
Gastrointestinal	Increased DGLA levels in Crohn’s disease	[29]
diseases	Increased DGLA levels in coeliac disease	[30]
Arthritis	Increased DGLA levels in rheumatoid arthritis	[31,32,33]
	DGLA exposure reduces synovial cell proliferation	[34]
	GLA supplement reduces rheumatoid arthritis symptoms	[8]
Bronchial asthma	Increased DGLA levels in disease cases	[35]
	Inverse relationship with lung function parameters	[36]
	GLA supplement reduces clinical symptoms	[37]
Atopic dermatitis	Reduced GLA and DGLA levels in disease cases	[38]
	GLA and DGLA supplements improve clinical signs	[38,39]
Cancers	Anti-proliferative effects of GLA and DGLA	[40]

HOMA-IR = homeostatic model assessment of insulin resistance.

Regarding cardiovascular diseases, apolipoprotein E-deficient mice fed with a DGLA supplement in their diet for 6 months showed a significant reduction in the development of atherosclerosis that may have been mediated via PGE_1_ [22]. In humans, *iv* PGE_1_ decreased the accumulation of radiolabeled apo B-containing lipoproteins in large arteries and the vascular cholesterol content [41]. In vitro, DGLA has attenuated pro-inflammatory gene expression in macrophages, chemokine-driven monocytic migration, macrophage foam cell formation, endothelial cell proliferation, and VSMC migration, as well as improved mitochondrial function [42]. GLA and PGE_1_ were also reported to exhibit anti-atherogenic effects. In patients with acute cardiovascular disease, low serum levels of DGLA and low DGLA/ARA ratios could predict poor long-term prognosis, especially in patients with acute decompensated heart failure [23]. In a similar way, low levels of DGLA in serum PLs were associated with an increased risk of death in elderly patients with a recent myocardial infarction [24].

In hepatic diseases, both non-alcoholic fatty liver disease (NAFLD) and non-alcoholic steatohepatitis were characterized by elevated concentrations of DGLA in total plasma lipids [25]. This could be due to increased ∆6-desaturase activity, as GLA levels also increased, whereas LA and ARA did not clearly respond. Similarly, higher serum concentrations of GLA and DGLA and higher ∆6-desaturase activities were associated with an increased risk for NAFLD, and higher ∆5-desaturase activities with a reduced risk [26]. Matsuda et al. [27] also agree that high serum DGLA levels or low ∆5-desaturase activities would be a strong predictor for hepatic steatosis. Interestingly, higher maternal plasma DGLA concentrations during pregnancy were associated with childhood liver fat accumulation at the age of 10, especially among boys [28]. This suggests that maternal n-6 PUFA balance could be of importance for the future well-being of the offspring, and disturbed PUFA metabolism may predispose to risks of adverse cardio-metabolic consequences later in life. A similar association was also observed between maternal plasma DGLA concentrations during pregnancy and total body fat mass percentages and abdominal preperitoneal fat mass areas at the age of six [21].

Inflammatory diseases of the gastrointestinal tract can also be characterized by altered n-6 PUFA metabolism. Patients with Crohn’s disease had elevated serum DGLA concentrations but decreased ARA levels compared to healthy controls [29]. Coeliac disease increased DGLA levels in erythrocyte membranes, while LA decreased and ARA was not significantly affected [30]. The reduced ARA/DGLA ratios suggest increased ∆6-desaturation, elongation, and/or decreased ∆5-desaturation. The n-6 PUFA metabolism was not completely restored by 12 months of gluten-free diet intervention. Regarding cancers, DGLA can have anti-proliferative properties, but n-6 PUFAs may also contribute to inflammation-driven tumorigenesis. Anti-cancer effects could be mediated through enhanced lipid peroxidation and synthesis of free radicals, PGE_1_, and 15-HETrE [40]. In colorectal carcinoma, the proportions of DGLA increased with parallel decreases in LA in cancerous tissue compared to adjacent normal tissue [43]. Thus, n-6 PUFA signatures could have potential as prognostic markers in malignancies. Regarding cancers in general, Pang et al. [44] suggested that ∆5-desaturase activity/expression may be an independent prognostic factor, although the mechanistic basis of this remains controversial. ∆5-Desaturase could aggravate cancer progression via mediating ARA/PGE_2_ production from DGLA, resulting in the activation of EP-receptors, inflammatory pathways, and immunosuppression. On the other hand, ∆5-desaturase may also prevent cancer progression via activated iron-dependent cell death (ferroptosis). Inhibition of ∆5-desaturation could shift the DGLA peroxidation pattern from generating ARA to a distinct anti-cancer free radical byproduct, 8-hydroxyoctanoic acid (8-HOA), resulting in the activation of apoptosis pathway and the suppression of cancer cell survival, proliferation, migration, and invasion (Figure 2). Accordingly, the knockdown of ∆5-desaturase along with DGLA treatment inhibited both motility and invasiveness of human colon cancer cells [45].

In inflammatory joint diseases, a pattern of decreased LA, increased DGLA, and stable ARA was documented for the serum PLs of RA patients [31]. Navarro et al. [32] also reported elevated DGLA in plasma and plasma PLs, whereas LA or ARA did not increase in RA. These findings support the view of stimulated ∆6-desaturation and elongation and slower ∆5-desaturation in chronically inflamed conditions. However, there were no changes in the n-6 PUFA profiles of RA synovial fluid (SF), which is in direct contact with the diseased synovial membrane and articular cartilage, compared to OA SF [32]. Our recent data show elevated DGLA and ARA proportions in RA synovium in comparison to the less inflamed OA synovial membrane, but similar LA and GLA percentages [33]. The anti-proliferative influence of DGLA has also been demonstrated in synoviocytes, whose aggressive proliferation is the hallmark of RA, leading to pannus formation as well as cartilage and bone degradation in synovial joints [34]. DGLA suppressed the proliferation of human adherent synovial cells compared to cultures grown with ARA, possibly due to increases in PGE_1_ production and cellular cAMP concentrations. DGLA and its derivative PGE_1_ also suppressed the expression of collagenase [46] and the release of matrix metalloproteinase-1 in synoviocytes [47] under inflammatory conditions. This is relevant, as different metalloproteinases participate in the destruction of the extracellular matrix of articular cartilage in OA and RA. In vivo, the supplementation of RA patients with GLA-rich oils, especially borage seed oil, resulted in promising improvements in tender/swollen joint counts and pain in randomized controlled trials [8].

Regarding respiratory diseases, the data are complex. Plasma PL DGLA levels had a positive association with asthma in young adults [35], and serum PL DGLA showed a negative association with lung function parameters, but only in men [36]. Interestingly, DGLA was elevated in bronchoalveolar lavage fluid (BALF), its cell-free supernatant, and extracellular vesicle (EV) fraction in the equine model of spontaneous asthma [48], while DGLA levels were reduced in the lungs of asthmatic mice sensitized by house dust mites [49]. DGLA may affect the pathogenesis of asthma via altered production of PGE_1_ that has beneficial effects on lung function by promoting bronchodilation [50] and by relaxing bronchial smooth muscle [51], or of ARA-derived cysteinyl LTs that are potent inducers of bronchospasm and airway inflammation [35]. Furthermore, PGE_2_ from ARA increases the relaxation of airway smooth muscle and inhibits the release of mast cell mediators and the recruitment of inflammatory cells [7]. This highlights the potentially multi-directional effects of the altered conversion of DGLA to ARA in respiratory disorders. To the best of our knowledge, the effects of dietary or inhalatory DGLA on lung function have not been in the focus of studies on respiratory diseases, while the results of dietary interventions with n-3 PUFAs on asthma control have not been encouraging [7]. This indicates that alternative n-6 PUFA-based treatments should also be investigated if lipid-based therapies for asthma continue to be considered viable supplements to conventional bronchodilators and inhaled corticosteroids.

Disturbed DGLA metabolism has also been connected to skin diseases. Patients with atopic dermatitis have lower circulating GLA and DGLA levels, which probably results from impaired ∆6-desaturase activities [38]. Peroral intake of GLA or GLA-rich oils can improve atopic dermatitis, but a lot of controversy remains regarding the interpretation of earlier literature. Still, supplementation with, for instance, GLA-rich evening primrose oil increased the plasma levels of GLA and DGLA in patients with atopic dermatitis and induced an improvement of clinical signs [39]. It is possible that a part of the patient population displays either non-compliance or failure of proper absorption or metabolization of GLA [52]. As their erythrocyte DGLA levels are not elevated by borage oil, their eczema would not benefit from dietary GLA, either. Currently, the role of GLA/DGLA in atopic dermatitis remains indefinite and continued research is warranted to study the connections between n-6 PUFAs and skin diseases, such as acne [53,54].

Some of the inflammatory diseases discussed above are age-associated, which would make their connections to n-6 PUFAs even more complex. The circulating concentrations of GLA, DGLA, and ARA can decrease in age-associated diseases simultaneously with a gradual increase in the pro-inflammatory state of the body [3]. These changes may partly derive from the decreased activities of desaturases, whereas COX-2 activity increases, and there are also alterations in the activities of LOX enzymes that have been documented with aging. These can lead to modifications at the levels of several n-6 and n-3 PUFAs and, subsequently, to increased production of PGE_2_ and LTB_4_/LTE_4_ and decreased formation of LXA_4_ and other SPMs.

To sum up, there are several inflammatory conditions, such as T2D, hepatic lipidosis, and Crohn’s disease, that exhibit elevated DGLA levels in circulation, and high DGLA could predict the risk for future metabolic syndrome or steatosis (Table 1). In contrast, there are indications that low DGLA levels can be associated with heart failure and complications of T2D. There also exist data that GLA administrations could induce beneficial effects on conditions including RA and atopic dermatitis. A defect in the activity of ∆6- and/or ∆5-desaturases, leading to either decreased or increased DGLA levels, may be a factor in the initiation and progression of certain inflammatory diseases.

## 4. Potential Applications of DGLA to the Prevention and Treatment of Inflammatory Conditions

When considering if DGLA could be used as a therapeutic agent in the medical conditions exemplified above, several aspects have to be taken into consideration. (i) Are there readily available products either containing purified DGLA or with a high DGLA content or can such products be developed in a practical manner? (ii) As DGLA can be associated with both disease amelioration and increased risk of particular disorders, how should we choose the conditions that would most probably benefit from DGLA therapy or from decreasing the body’s DGLA levels by enzyme inhibitors or nutritional manipulation? (iii) Is it possible to construct an effective and safe dosage of DGLA at this point without significant risks or side effects?

### 4.1. Potential Dietary Sources of DGLA

In order to develop practical and cost-effective products either of purified DGLA or high in DGLA content, the possibilities include DGLA production in, for instance, fungal cultures. In the case of FA mixtures, the overall FA profile of a product, such as its n-3/n-6 PUFA ratio, should be health-promoting. For medical use, the DGLA proportion or concentration of a product should obviously be reported in a manner necessary for therapeutic products (for instance, wt-% or mg/volume). Furthermore, a requirement for the peroral use of DGLA as a remedy is that it would actually cause measurable increases in the body’s DGLA content, which does take place making this route of administration feasible [55].

Producing DGLA per se is becoming a possibility in bioreactors by using physiological manipulation of micro-organisms [56]. With specific culture media and an engineered *Pythium* Δ6-desaturase and Δ6-elongase overexpressing *Aspergillus oryzae* strain (a filamentous fungal organism used in fermentation), the DGLA content of the produced FAs can be >20% with GLA and LA production also at a high level. Another model studied is a ∆5-desaturase-defective mutant of *Mortierella alpina*, a soil fungus, in which DGLA can represent 43% of total FAs with a very low ARA level [57,58]. Other potential methods to produce DGLA for therapeutic use could be cultures of, for instance, genetically engineered microalgae [59] and *Saccharomyces cerevisiae* [60]. Eventually, these approaches could make the production of DGLA-enriched oils technically and commercially feasible.

Another class of oils derives from borage, blackcurrant, and evening primrose seeds, which, however, are rich in GLA [1,61]. In this case, it should be emphasized that DGLA supplementation per se is not a requirement for increased body DGLA concentrations, as GLA is known to increase, e.g., serum and neutrophil DGLA levels [62]. GLA-containing oils are being actively used and marketed as natural products that would have health benefits with hitherto little carefully examined justification. For instance, the search term “GLA oil” + purchase yields approximately 5000 hits on the Google search engine. Although oils are principally consumed perorally or topically, an emerging method to transfer DGLA into organisms could be to use cell-derived EVs as carriers. The lipid bilayer of EVs has been shown to contain small proportions of DGLA, and its levels can vary depending on disease state. For instance, DGLA proportions increased in BALF EVs in severe equine asthma [48], and EVs could also have a role in the entry of this PUFA into SF [63]. It may be possible to increase DGLA proportions in EV membrane lipids by in vitro manipulations of parent cells with PUFAs.

### 4.2. Therapeutic Potential of DGLA Supplementation or Suppression of DGLA Metabolism

As reviewed above (Table 1), several disease states are characterized by abnormally low DGLA levels in the body (atopic dermatitis, cardiac disease), while some others are featured with elevated DGLA levels (NAFLD, T2D). Next, we discuss the possibilities (i) to increase body DGLA, with FA supplements or by inhibiting ∆5-desaturation, and (ii) to decrease endogenous DGLA production by inhibiting ∆6-desaturation.

The most extensively studied application of DGLA is probably the peroral administration of GLA oils to treat atopic dermatitis. The potential of GLA and, through metabolic conversion, DGLA as a remedy for this skin condition was noted decades ago, but there was no significant difference in the improvement of symptoms from placebo [64]. In animal studies, the findings were more promising including the prevention of the development of atopic dermatitis by peroral DGLA [65]. More recent human studies still lack any robust and unequivocal demonstration of the efficacy [53]. Although results from clinical studies indicate that GLA preparations seem to be safe with few side effects, and also show some improvement of atopic dermatitis symptoms, Bamford et al. [9] concluded in a Cochrane review that oral borage or evening primrose oils did not have beneficial effects on eczema and any improvement was similar to placebo. Research is still being carried out, and by using the search terms “DGLA” or “dihomo gamma linolenic acid”, the ClinicalTrials.gov database https://clinicaltrials.gov/ct2/results?cond=&term=DGLA&cntry=&state=&city=&dist= (accessed on 11 January 2023) lists 15 trials, of which six involve atopic dermatitis.

Another potential condition for DGLA supplementation could be the respiratory disease in the continuum that includes atopic dermatitis, that is, bronchial asthma but, also here, existing results are not too encouraging, and the same seems to be true for other FA therapies [7]. Although asthma patients receiving GLA (2 g/day) in borage oil experienced significantly increased DGLA concentrations in neutrophil PLs and serum, there was no significant improvement in clinical parameters [66]. Metabolically, GLA elevated 15-HETrE in neutrophils with a parallel suppression of LTB_4_ production. In a more recent experiment, GLA supplementation was able to reduce asthma symptoms but did not have measurable effects on inflammatory markers [37]. Thus, it seems that while many studies attain an increase in body DGLA levels, actual measurable clinical benefits should also be observed in the future in order to recommend DGLA as a remedy for asthma or other conditions. As n-6 and n-3 PUFAs are competitively metabolized by the same set of desaturase, elongase, and oxygenase enzymes [67], it has also been suggested that combined GLA and n-3 PUFA (EPA, DHA) supplementation could suppress inflammatory processes most effectively [54,68].

In metabolic syndrome, the picture is obviously different, as there is a positive association between T2D and DGLA levels, while the relationship can be inverse with complications of T2D (Table 1). In this case, DGLA therapies cannot be recommended but, in combination with other therapeutic interventions, Serhiyenko and Serhiyenko [69] suggested that *α*-lipoic acid, DGLA, n-3 PUFAs, and their simultaneous administration with aldose reductase inhibitors could be considered as potential treatments for T2D-associated cardiac autonomic neuropathy. Still, as DGLA levels are increased with obesity [11] and could, thus, be associated with the inflammatory phenomena that characterize the condition, DGLA supplementation cannot be recommended to counteract the low-grade inflammation in obesity-associated disorders at the moment. However, decreasing the body DGLA levels with, e.g., selected PUFA supplementation [70] could be useful in these cases and, furthermore, DGLA may have utility as a biomarker of increased inflammatory risk. The issue remains complicated, as low levels of DGLA were previously associated with higher mortality caused by myocardial infarction [24]. In a Cochrane review [71], Hooper et al. did not find suitable studies targeting DGLA as a therapeutic agent, but they surmised that specific trials with DGLA and ARA would not necessarily be required as these n-6 PUFAs can be synthesized from GLA, albeit at low levels. Generally, the effects of n-6 PUFAs on cardiovascular events or deaths were negligible even though they may reduce the risk of heart attacks, but high-quality studies were absent. Thus, evidence for the cardiovascular benefits of DGLA remains meager, and it should also be remembered that coronary disease, obesity, NAFLD, and T2D can all be associated with one another, which makes it very difficult at this stage to assess the overall benefits or potential adverse effects of DGLA supplementation in this disease continuum. However, it also seems that no dramatic therapeutic benefits could be expected.

Instead of using dietary means to modify circulating DGLA levels, metabolic interventions could be utilized to either decrease the formation of DGLA from GLA or to increase its concentrations (Figure 2). Increasing DGLA levels with the inhibition of ∆5-desaturase would not only have effects on DGLA and its PG derivatives but would also decrease the amount of ARA and, consequently, its lipid mediators, which could be another promising way to apply DGLA-related pathways to treatment. In addition to potentially increasing the amount of DGLA-derived anti-inflammatory derivatives, it has been suggested that the inhibition of ∆5-desaturase could also promote DGLA peroxidation leading to the production of 8-HOA by COX-2, and this byproduct would decrease cancer cell viability and proliferation [44]. Yet, any trials with ∆5-desaturase inhibitors, such as curcumin and sesamin [40], would have to take into consideration the opposing effects that DGLA seems to have on different malignancies. For instance, ∆5-desaturase may induce protective effects against non-small-cell lung cancer but be associated with poorer prognosis in bladder cancer [44]. Thus, the transition from in vitro studies to patient trials will require a significant amount of additional research to yield specific hypotheses and to ensure patient safety. In addition to cancers, it is plausible that ∆5-desaturase inhibition could eventually be tested in other disease states that exhibit decreased DGLA levels and/or excess ARA compared to control. A small-scale study on bronchial asthma, for instance, indicated that while the forced expiratory volume in one second improved when curcumin was combined with conventional medication, the reduction in the severity of asthma symptoms did not differ from standard therapy [72].

The possibility of ∆6-desaturase inhibitors to decrease DGLA levels has been examined especially in malignant diseases, at this stage almost exclusively in vitro. Of these inhibitors, SC-26196 is being actively studied [73]. He et al. [74] tested this compound in mice injected with melanoma and lung cancer cells and observed reduced tumor growth, probably triggered by the decrease in ARA and, consequently, its derivatives, such as PGD_2_, PGE_2_, 12-hydroxyeicosatetraenoic acid (HETE), and 15-HETE. The more DGLA and ARA there were in comparison to LA, the larger the tumors tended to be. This does not suggest direct tumorigenic effects of DGLA but its role as an intermediate product in the pathway producing tumor growth-enhancing molecules. SC-26196 also sensitized glioblastoma cells and tumors to radiotherapy, thus, enhancing the treatment outcome of this very aggressive malignancy of the central nervous system with some promise for the treatment of resistant tumors [75]. However, it seems that the inhibition of ∆6-desaturation is not necessary to obtain beneficial effects from the suppression of the GLA→DGLA→ARA pathway. Fussbroich et al. [49] observed a deficiency of DGLA and a surplus of ARA in the lungs of asthmatic mice. Supplementation with long-chain PUFAs (EPA, DHA, GLA, and 18:4n-3) caused reductions in ARA and corrected the DGLA levels to those of the control mice. Although no clinical variables were examined, the authors concluded that this long-chain PUFA combination could prove to be beneficial in limiting the inflammatory processes in the lungs.

To conclude, there are different strategies targeting DGLA and ARA production and, thus, the concentrations of their pro- and anti-inflammatory derivatives. These seem to be especially promising in some cancers and perhaps also in asthma, but clinical data remain scarce and further well-designed studies should be actively promoted.

### 4.3. Potential Risks of Dietary DGLA Administration

To become a viable treatment option, a remedy should be at least as effective as existing medical substances and not cause higher risks than those of present alternatives. When FA therapies are designed, it should be remembered that modifying the body’s n-3/n-6 PUFA balance with GLA or DGLA could obviously cause an increase in the downstream products of these PUFAs [2]. These include potentially pro-inflammatory lipid mediators, especially if supplementing with n-6 PUFAs alone, which could probably be avoided with supplements combining GLA or DGLA to n-3 PUFAs. Unlike previously assumed, there would be no elevated risk of clinically significant bleeding caused by n-3 PUFA consumption [76].

Peroral DGLA as a supplement seems to be well tolerated with no significant side effects in humans [55]. When 20 volunteers were assigned either into a placebo or DGLA group (administered as *M. alpina* oil corresponding to 450 mg DGLA/day) to determine if peroral supplementation with DGLA would increase its blood concentrations without significant side effects or deleterious changes in health parameters, no differences were observed in hematological or biochemical variables, coagulation parameters, or blood pressure between the groups. Although the exposure time was relatively short (4 weeks), the results indicate that the risks of DGLA consumption would be small. Still, transient gastrointestinal side effects have been reported [53].

Preliminary results from two clinical studies at the ClinicalTrials.gov database https://clinicaltrials.gov/ct2/results?cond=&term=DGLA&cntry=&state=&city=&dist= (accessed on 11 January 2023) also state that serious adverse effects were rare and equal in both placebo and DGLA groups (2 g of patented DGLA preparation DS107/day). Gastrointestinal side effects have also been observed with GLA preparations, such as evening primrose and borage oils, and there are some hitherto mostly anecdotal warnings of prolonged consumption having the potential to cause either thrombosis or, in contrast, bleeding when used with anti-thrombotic medication [9].

As pharmaceutical ∆6- and ∆5-desaturase inhibitors have been tested mostly on experimental animal models, data about their risks and side effects remain scarce. An obvious concern is the potential deficiency of ARA that could be caused by effective ∆6-desaturase inhibition. However, this would be quite implausible as the ARA content of Western diets is high and, therefore, body ARA would not be depleted even if its endo-genous production were reduced [74]. Taking into consideration the essential nature of ARA derivatives in organisms, however, it would be necessary to carefully assess any long-term effects of ∆6-desaturase inhibition on the eicosanoid system. Similarly, in addition to decreasing the overall ARA levels, ∆5-desaturase inhibition has been shown to cause a specific decrease in the ARA concentrations in mouse brains [77]. This phenomenon could cause potentially significant side effects, as endocannabinoids are ARA derivatives, and lowering the brain ARA concentration could cause neuropsychiatric effects, including depression, via altered signaling of cannabinoid receptor type 1.

In summary, the consumption of DGLA for moderate periods of time seems to pose no significant risks but, as extensive long-term studies on human patients are still lacking, this remains to be determined for ∆6- and ∆5-desaturase inhibitors.

## 5. Conclusions

Despite its low proportions in mammalian tissues, DGLA per se and its derivatives are emerging as important mediators of inflammation with potential significance in particular disease conditions. However, direct medical applications of specially purified DGLA preparations seem unconvincing at the moment. Although DGLA therapies, in particular monotherapies, do not seem to be very promising, the manipulation of metabolic pathways with ∆6- and ∆5-desaturase inhibitors or combinations of long-chain PUFA supplements also containing n-3 PUFAs could provide a way to modify the body DGLA content in conditions of inflammation and malignancies. Existing research data mostly consist of associations of body DGLA levels with various disease states, but the significant therapeutic effects of DGLA administrations remain modest, even at best. Despite DGLA supplements being unpromising as a curative or ameliorating therapy, the position of DGLA at a pivotal point of several metabolic pathways leading to anti-inflammatory derivatives or, via ARA to pro-inflammatory mediators, makes this n-6 PUFA an intriguing research subject regarding the possibilities of influencing these synthesis reactions towards an anti-inflammatory direction, especially in cancer therapies. Instead of DGLA supplements, the manipulation of DGLA and its derivatives by ∆6- and ∆5-desaturase inhibitors could be a more promising form of future therapy.

## Data Availability

Not applicable.

## Abbreviations

ARA	arachidonic acid
BALF	bronchoalveolar lavage fluid
COX	cyclooxygenase
DGLA	dihomo-*γ*-linolenic acid
DHA	docosahexaenoic acid
ELOVL	fatty acid elongase
EPA	eicosapentaenoic acid
EV	extracellular vesicle
FA	fatty acid
FADS	fatty acid desaturase
GLA	*γ*-linolenic acid
HETE	hydroxyeicosatetraenoic acid
15-HETrE	15-(S)-hydroxy-8,11,13-eicosatrienoic acid
8-HOA	8-hydroxyoctanoic acid
HOMA-IR	homeostatic model assessment of insulin resistance
LA	linoleic acid
LOX	lipoxygenase
LT	leukotriene
LXA_4_	lipoxin A_4_
NAFLD	non-alcoholic fatty liver disease
OA	osteoarthritis
PG	prostaglandin
PL	phospholipid
PUFA	polyunsaturated fatty acid
RA	rheumatoid arthritis
SF	synovial fluid
SPM	specialized pro-resolving mediator
T2D	type 2 diabetes
VSMC	vascular smooth muscle cell
